# Validity and Reliability of Wearable Sensors for Continuous Postoperative Vital Signs Monitoring in Patients Recovering from Trauma Surgery

**DOI:** 10.3390/s24196379

**Published:** 2024-10-01

**Authors:** Rianne van Melzen, Marjolein E. Haveman, Richte C. L. Schuurmann, Kai van Amsterdam, Mostafa El Moumni, Monique Tabak, Michel M. R. F. Struys, Jean-Paul P. M. de Vries

**Affiliations:** 1 Department of Surgery, Division of Vascular Surgery, University Medical Center Groningen, University of Groningen, 9713 GZ Groningen, The Netherlandsj.p.p.m.de.vries@umcg.nl (J.-P.P.M.d.V.); 2 Department of Anesthesiology, University Medical Center Groningen, University of Groningen, 9713 GZ Groningen, The Netherlands; 3 Department of Surgery, Division of Trauma Surgery, University Medical Center Groningen, University of Groningen, 9713 GZ Groningen, The Netherlands; 4 Department of Biomedical Signals and Systems, University of Twente, 7500 AE Enschede, The Netherlands

**Keywords:** telemonitoring, validity, accuracy, vital signs, wearable sensor, surgical ward

## Abstract

(1) Background: Wearable sensors support healthcare professionals in clinical decision-making by measuring vital parameters such as heart rate (HR), respiration rate (RR), and blood oxygenation saturation (SpO_2_). This study assessed the validity and reliability of two types of wearable sensors, based on electrocardiogram or photoplethysmography, compared with continuous monitoring of patients recovering from trauma surgery at the postanesthesia care unit. (2) Methods: In a prospective observational study, HR, RR, SpO_2_, and temperature of patients were simultaneously recorded with the VitalPatch and Radius PPG and compared with reference monitoring. Outcome measures were formulated as correlation coefficient for validity and mean difference with 95% limits of agreement for reliability for four random data pairs and 30-min pairs per vital sign per patient. (3) Results: Included were 60 patients. Correlation coefficients for VitalPatch were 0.57 to 0.85 for HR and 0.08 to 0.16 for RR, and for Radius PPG, correlation coefficients were 0.60 to 0.83 for HR, 0.20 to 0.12 for RR, and 0.57 to 0.61 for SpO_2_. Both sensors presented mean differences within the cutoff values of acceptable difference. (4) Conclusions: Moderate to strong correlations for HR and SpO_2_ were demonstrated. Although mean differences were within acceptable cutoff values for all vital signs, only limits of agreement for HR measured by electrocardiography were considered clinically acceptable.

## 1. Introduction

Healthcare support through technology, such as wearable sensors, emerges as a pivotal component in addressing the challenges of staff shortages and increasing demand for care. The use of wearable sensors can decrease nurse workload and improve feelings of safety for patients in perioperative care by continuously measuring vital signs, such as heart rate (HR), respiration rate (RR), blood oxygenation saturation (SpO_2_), and temperature [1,2]. Nowadays, nurses in the hospital manually perform these measurements intermittently, which is time-consuming [3].

Wearable sensors can support healthcare professionals in detecting the deterioration of patients’ vital signs. This technology also ensures that healthcare providers have continuous access to trend data on vital signs, which can contribute to improved clinical decision-making [4,5,6]. In light of this, postsurgical patients, who are often at risk of complications, can be closely monitored [7]. Rowland et al. showed that continuous monitoring of adult surgical ward patients was associated with a reduced risk of in-hospital mortality and intensive care unit (ICU) admissions [8]. Although the potential of continuous monitoring of vital signs is increasingly acknowledged, the implementation of wearable sensors in the surgical ward is not routinely integrated into standard care.

In the context of the nonadoption, abandonment, scale-up, spread, and sustainability framework, vital signs measured by a wearable sensor should be compared against the standard clinical reference monitoring in a clinical environment involving the target patients [9,10]. Evidence is scarce in this particular field. However, the validity and reliability of wearable sensors have been investigated in other contexts and patient groups, such as a controlled setting involving healthy volunteers or at a surgical ward, compared with intermittent nurse measurements [11,12]. In addition, a variety of wearable sensors are commercially available with different features and technology for measuring vital signs. As a consequence, choosing a wearable sensor for implementation in a surgical ward can be challenging.

Measurements derived from electrocardiography (ECG) and photoplethysmography (PPG) are the most common techniques in wearable sensors. Therefore, the objective of this study was to assess the concurrent validity and reliability of two types of wearable sensors for vital signs monitoring, one based on ECG and one on PPG, compared with continuous monitoring of patients recovering from trauma surgery at the postanesthesia care unit (PACU).

## 2. Materials and Methods

### 2.1. Design

This single-center observational pilot study investigated the feasibility and applicability of wearable sensors at the University Medical Center Groningen (UMCG) (Postoperative continuous telemonitoring; an observational study [TRUST study], ISRCTN75034369). This study assessed data collected through continuous monitoring with 2 types of wearable sensors against standard clinical monitoring practice in the PACU as a reference. The protocol was approved by the UMCG Ethical Committee and was performed according to the Declaration of Helsinki.

### 2.2. Participants

Patients aged >18 years and admitted for trauma surgery between September 2023 and April 2024 with an expected hospital stay of at least 48 h were invited to participate in this study. Consecutive eligible patients scheduled for surgery received an information letter during the preadmission consultation at the outpatient clinic and were asked for informed consent during admission. For nonelective admissions, eligible patients received the information letter on the surgical ward. The subsequent day, patients provided informed consent and received instructions from the research nurse on using wearable sensors in the surgical ward. Trauma patients needing urgent surgery before consent could be signed were excluded. Participants were excluded if they were mentally incapable of participating or if the nature of the injury prevented them from wearing the sensors.

### 2.3. Data Collection

Data were collected from the 2 wearable sensors during patients’ postoperative stay at the PACU. VitalPatch (MediBioSense, Doncaster, UK) is a CE Class IIa-certified sensor enabling noninvasive ECG-based measurements of HR (variability), RR, body temperature, and physical activity (step count, and type) at a frequency of once every 4 s. Radius PPG (Masimo, Irvine, CA, USA) is a CE Class IIa-certified sensor enabling noninvasive PPG-based measurement of HR (hence actually pulse rate), RR, and SpO_2_ at a frequency of once per 2 s. Body temperature was additionally measured using the Radius T (Masimo, Irvine, CA, USA) at a frequency of once every 60 s. Data were stored locally for both devices, taking time synchronization into account. Philips IntelliVue patient monitors (Philips, Amsterdam, The Netherlands) were used as reference monitoring during the patients’ stays at the PACU.

This reference uses ECG for HR and RR detection and PPG for SpO_2_. Body temperature was measured by a probe through the urinary catheter when present, according to standard care, and is widely accepted as an accurate measurement of central body temperature [13]. Data were stored in the electronic health record (EHR) with a frequency of once every 15 s. After study completion, data were exported from the EHR and stored on the local research drive according to the data management plan.

### 2.4. Protocol

**Figure 1:**
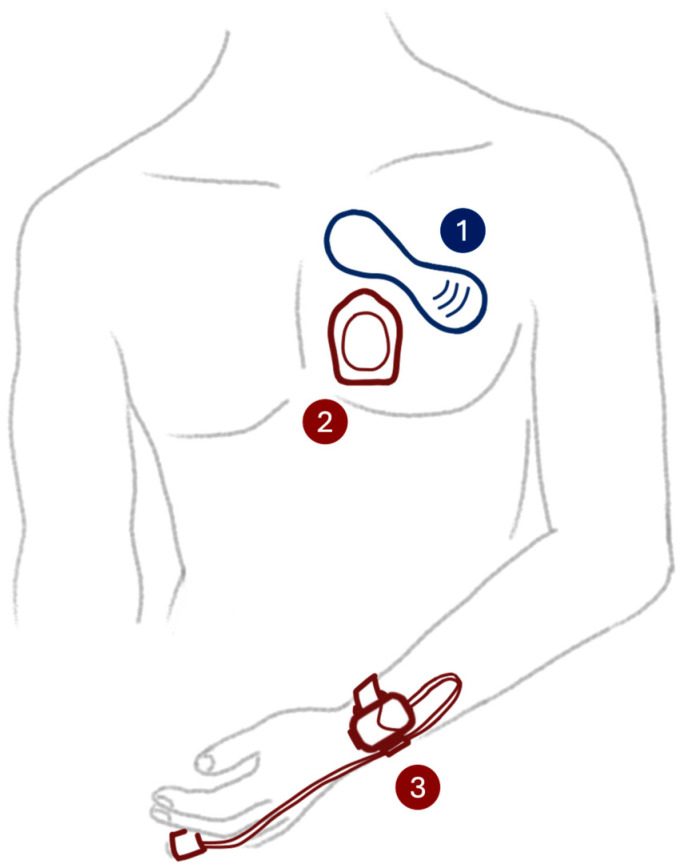
Placement of the wearable sensors on the participant’s body: (1) VitalPatch for HR and RR based on ECG and temperature; (2) Masimo Radius T for temperature; and (3) Masimo Radius PPG measuring HR (pulse rate), RR and SpO_2_ based on PPG.

Directly after postoperative admission at the PACU, the wearable sensors were connected to the patient by the research nurse following the manufacturer’s instructions, as illustrated in Figure 1. The VitalPatch and Radius T were positioned on the patient’s chest, and the Radius PPG was positioned on the patient’s wrist with a sensor on the fingertip. The VitalPatch was connected to the accompanying tablet application, and a motion calibration was performed subsequently according to the instructions for use. A temperature calibration using the reference core temperature was requested 30 min after application of the VitalPatch. The Radius PPG and Radius T were connected to a bedside monitor (Rad97, Masimo, Irvine, CA, USA) via Bluetooth. Patients were instructed to wear the sensors continuously during their stay at the PACU. Patients and healthcare professionals had no access to the study data and did not receive any feedback from the monitor. This study had no impact on the surgical procedure or postsurgical treatment, all of which were performed in accordance with the standard of care.

### 2.5. Statistical Analyses

Vital signs were analyzed separately per sensor and compared with the reference PACU monitoring. For VitalPatch, HR, RR, and body temperature were analyzed. For Radius PPG and Radius T, HR, RR, SpO_2_, and body temperature were included in data analyses. Time synchronization was guaranteed because all devices used the institution’s network-synchronized computer time, which was confirmed by data visualization. Data were preprocessed before data analyses by removing the first minute of measurements for each vital sign. For all patients, data were continuously recorded until discharge from the PACU.

Data points for each vital sign were paired using nearest neighbor interpolation between the measurements of the wearable sensor and the reference. The maximum time shift in the pairing was selected as the lowest sample time for each wearable, which was 4 s for VitalPatch, 60 s for Radius T, and 2 s for Radius PPG.

The analyses used 2 different sets of data pairs. For the first analysis, 4 random data pairs were selected from these preprocessed data. This demonstrated concurrent validity and reliability at any random point in time. For the second analysis, a median filter of 1 min was applied for each vital sign during the initial 30-min period. This period was chosen to use a similar number of data pairs per patient in the analyses. The 30 median data pairs were included in data analyses to take into account concurrent validity and reliability over time.

Descriptive statistics consists of the number of available data pairs, median values with interquartile ranges (IQR), and median absolute percentage error (MAPE) for both wearable sensors compared with reference measurements for each vital sign. Subsequently, for each set of data pairs, each of the wearable sensors was evaluated for concurrent validity and reliability for HR, RR, SpO_2_, and body temperature. To evaluate the validity of the wearable sensors, repeated-measures correlation coefficients were calculated for each vital sign using the rmcorr package from R 4.4.1 software (R Foundation for Statistical Computing, Vienna, Austria) [14]. A correlation coefficient of <0.5 was considered a weak, 0.5 to 0.7 a moderate, and 0.7 to 1.0 a strong relationship [15].

Reliability was assessed using Bland–Altman plots, mean differences, and 95% limits of agreement (LoA) for each vital sign. Bland–Altman analyses were adjusted for repeated measurements, accounting for the variance between measurement pairs as the sum of both between-subject and within-subject variances [16]. Cutoff values of the acceptable difference between the wearable sensor and reference measurements were used as prior hypotheses based on the modified Early Warning Score [17] and previously published work [9], being ±5 beats/min (bpm) for HR, ±2 breaths/min (brpm) for RR, ±0.5 °C for body temperature, and ±2% for SpO_2_. Data were processed and analyzed in MATLAB R2023b software (MathWorks, Inc., Natick, MA, USA).

## 3. Results

This study enrolled 60 patients. Patient characteristics are presented in Table 1. Two patients were excluded because the wearable sensors were not applied at the PACU due to logistical reasons. Of the 58 included patients wearing VitalPatch, data availability differed regarding all vital signs. HR data in nine patients were not available or only available for <5 min because of connection issues (n = 6) and in patients with data < 5 min due to delay caused by connection difficulties (n = 3). RR data in 15 patients were not available or had limited availability because of delayed motion calibration (n = 4), connection problems (n = 6), or patients with data < 5 min (n = 5). Body temperature data were not available for 28 patients because of delayed calibration or technical calibration problems (n = 6), connection problems (n = 10), patients with data < 5 min (n = 5), or unknown (n = 7).

Of the 58 patients, data for seven patients were not available from Radius PPG. The reasons were that a patient withdrew consent for the Radius PPG sensor (n = 1), and problems with the export of the data at 96 h caused the loss of data (n = 6). Data for Radius T were not available for 11 patients due to withdrawn consent for the Radius T sensor (n = 1), export problems of the data (n = 6), connection problems (n = 3), or because the Radius T was not applied (n = 1).

Reference monitoring data for HR and RR were available for all 58 patients. For body temperature, reference data by urinary catheter were available for 5 patients with VitalPatch and 12 patients with Radius T data. Table 2 reports the availability of data pairs for each vital sign per patient for VitalPatch, including the median (IQR) values and MAPE. The same results are presented in Table 3 for Radius PPG and T.

### 3.1. Concurrent Validity: Randomly Selected Pairs

Repeated-measures correlation coefficients between four randomly selected pairs of vital sign measurements per patient showed a moderate relationship between HR measured by VitalPatch and the reference monitor (R = 0.57; 95% CI; 0.45–0.67). The correlation coefficient for VitalPatch RR was weak (R = 0.08; 95% CI; 0.09–0.25).

For Radius PPG, validity analysis showed a moderate relationship with the reference monitor for HR (R = 0.60; 95% CI; 0.49–0.69). The correlation coefficient of Radius PPG for RR was weak (R = 0.20; 95% CI; 0.04–0.35). The correlation between Radius PPG and the reference monitor for SpO_2_ was moderate (R = 0.57; 95% CI; 0.45–0.67).

### 3.2. Concurrent Validity: 30-min Pairs

Repeated-measures correlation coefficients between median values of the first 30 min per vital sign per patient showed a strong relationship between HR measured by VitalPatch and the reference monitor, with a correlation coefficient of 0.85 (95% CI; 0.83–0.86). The correlation coefficient for VitalPatch RR was 0.16 (95% CI; 0.10–0.22), which is considered a weak relationship.

**Figure A1:**
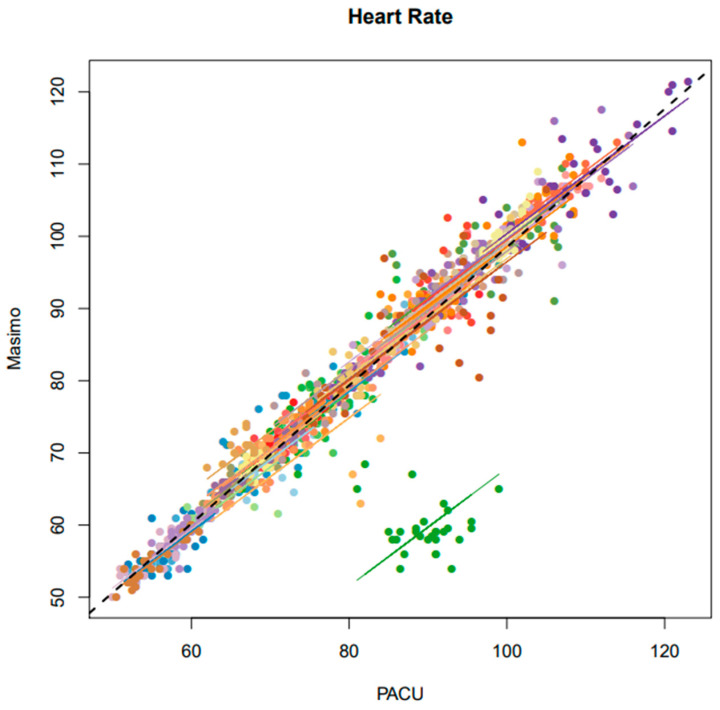
Scatterplot for the validity analysis of the Radius PPG compared with the reference monitor for heart rate (HR) measured in beats per minute (bpm). The green cluster demonstrates data from a patient in whom an unknown rhythm was detected by the reference monitor. The x-axis presents HR measured with the reference monitor at the postanesthesia care unit (PACU), and the y-axis represents HR measured by Radius PPG in bpm. The dots represent the data from each patient by a specific color. The lines represent the correlation coefficient per patient.

For Radius PPG, validity analysis showed a strong relationship with the reference monitor for HR (R = 0.83; 95% CI; 0.82–0.85). Noteworthy is a cluster of outliers associated with one patient in the scatter plot, as shown in Figure A1 in Appendix A. For Radius PPG, the correlation coefficient was weak for RR (R = 0.12; 95% CI; 0.07–0.17) and moderate for SpO_2_ (R = 0.61; 95% CI; 0.57–0.64).

### 3.3. Reliability: Randomly Selected Pairs

**Figure 2:**
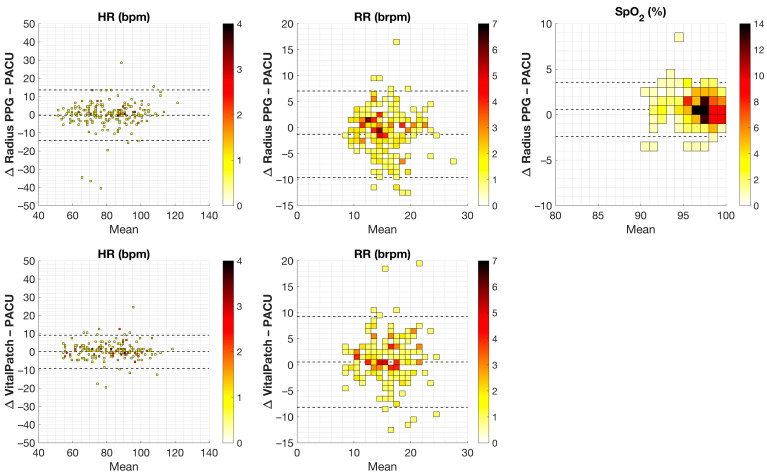
Bland–Altman plots for the Radius PPG vs. reference (upper row) and VitalPatch vs. reference (lower row) for the 4 randomly selected data pairs per patient for each vital sign: heart rate (HR) in beats/min (bpm); respiration rate (RR) in breaths/min (brpm); and blood oxygen saturation (SpO_2_) in percentage (%). The x-axis represents the mean, and the y-axis represents the difference (Δ) between both measurement pairs. The dotted lines represent the mean difference and 95% limits of agreement for repeated measurements. The heat map represents the number of pairs in the specific bin.

Figure 2 shows Bland–Altman plots per wearable sensor per vital sign with the mean difference and LoA for the four randomly selected data pairs for each patient. Table 4 shows the corresponding values for the mean differences and 95% limits of agreement. For VitalPatch versus reference monitoring, mean differences in HR and RR were within the predefined cutoff values of acceptable differences of ±5 bpm and ±2 brpm, respectively. However, the LoA were not.

Also, for Radius PPG, all of the mean differences were within the predefined cutoff values of acceptable difference. In addition to HR and RR, this was ±2% for SpO_2_. All parameters showed LoA outside the acceptable differences.

### 3.4. Reliability: 30-min Pairs

**Figure 3:**
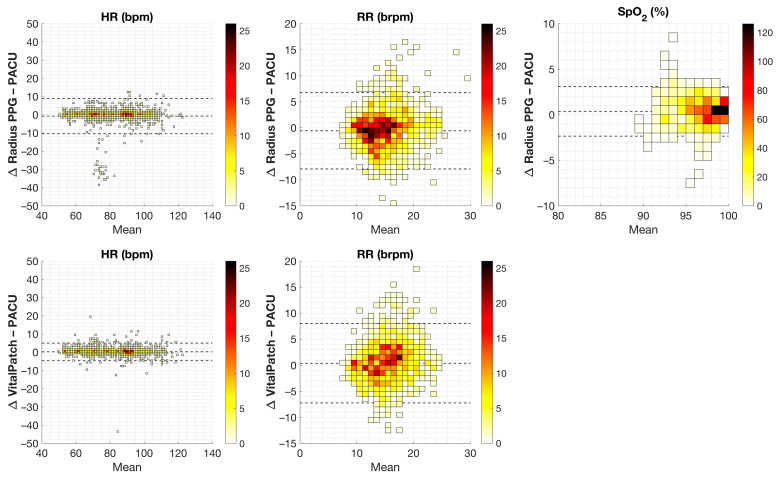
Bland–Altman plots for the Radius PPG vs. reference (upper row) and VitalPatch vs. reference (lower row) for the 30-min median value pairs per patient for each vital sign: heart rate (HR) in beats/min (bpm), respiration rate (RR) in breaths/min (brpm), and blood oxygen saturation (SpO_2_) in percentage (%). The x-axis represents the mean, and the y-axis represents the difference (Δ) between both measurement pairs. The dotted lines represent the mean difference and 95% limits of agreement for repeated measurements. The heat map represents the number of pairs in the specific bin.

Figure 3 shows Bland–Altman plots for each wearable sensor per vital sign with the mean difference and LoA for the 30-min median value pairs per patient shown in Table 4. For VitalPatch, both mean differences were within the predefined cutoff values of acceptable difference. For HR, the LoA were within the cutoff values of acceptable difference, whereas RR LoA were not.

For Radius PPG, mean differences in all vital signs were within the predefined cutoff values of acceptable difference. However, the LoA for all parameters were outside the acceptable differences. For HR, the same cluster of outliers, as described in 3.2, is visible in Figure 3.

## 4. Discussion

We assessed two types of wearable sensors for vital signs monitoring, one based on ECG (VitalPatch) and one on PPG (Radius PPG), compared with a clinical reference monitor as an important step toward implementation of continuous monitoring with wearable sensors on a surgical ward. Results of this study show that the ECG-based and PPG-based wearable sensors both have mean differences within acceptable cutoff values for measuring HR, RR, and SpO_2_. However, the LoA only for HR measured by ECG is clinically acceptable. Unlike HR and SpO_2_, this study could not demonstrate the validity of RR. The performance of the wearable sensors for body temperature could not be analyzed due to the absence of continuous reference measurements in clinical practice.

### 4.1. VitalPatch

For validity, the performance is strong for HR based on the analyses from the 30-min pairs and moderate for randomly selected data. We found a weak relationship for RR. The inaccuracy of RR in wearable sensors is often attributed to the location of the sensor or the influence of movement [9,11,18]. However, the VitalPatch was applied to the chest during this study, which caused the least movement, and the patients were in bed postoperatively. As a consequence, this study was not able to validate RR measurements for the VitalPatch. Simultaneously, the inaccuracy of nurses’ RR measurements in the surgical ward has also been documented [19,20]. This indicates the challenges regarding this parameter.

VitalPatch shows mean differences for HR and RR of close to 0. However, we considered vital signs acceptable if the LoA were within the previously hypothesized cutoff values. This applies to HR in the 30-min analysis. The results are in line with those of Breteler et al., confirming that applying a median filter results in smaller LoA [21]. Moreover, studies assessing wearable sensors against intermittent vital signs measurements found wider LoA [12,22]. These wearable sensors are not intended to provide intermittent measurements, which could explain the difference. However, this may also be a consequence of the increasing amount of data included using continuous measurements compared with intermittent measuring.

### 4.2. Radius PPG

**Figure A2:**
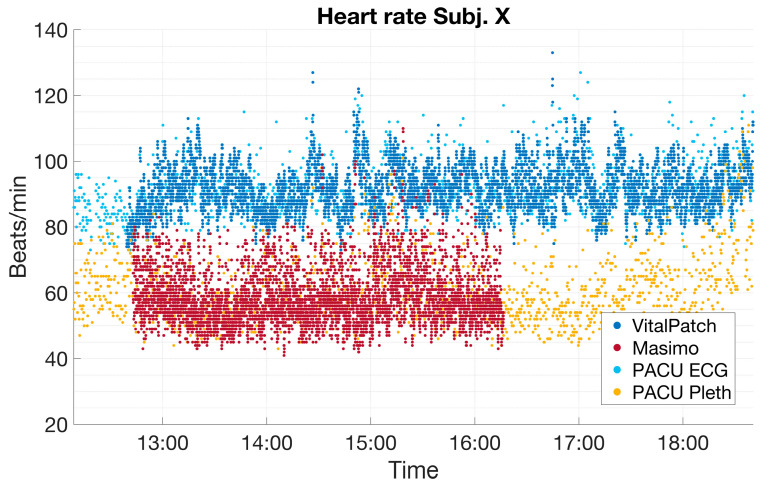
Heart rate (HR) in beats per min (bpm) of a patient demonstrating the comparison of Radius PPG HR (red) and HR measured by the photoplethysmography (Pleth) monitor at the postanesthesia care unit (PACU; yellow) versus HR measured based on electrocardiogram (ECG) by VitalPatch (dark blue) and ECG reference monitor at the PACU (light blue). The y-axis represents HR in bpm, and the x-axis represents time in hours.

For validity, the performance is strong for HR based on the analysis from the 30-min pairs and moderate for randomly selected data. Notable for the Radius PPG data for HR is the green cluster from a patient in whom an unknown rhythm was detected by the reference monitor (Figure A1 in Appendix A). Comparison of Radius PPG HR and HR measured by the PPG monitor at the PACU (standard care) shows a similar pattern that is different from the ECG data (Figure A2 in Appendix A). Breteler et al. also described a substantial variability of HR, which was observed during episodes of atrial fibrillation in five patients [23]. The wearable sensor used in the study of Breteler et al. is similar to the Radius PPG. The patient with this phenomenon in our study was also diagnosed with atrial fibrillation. Similar to the ECG-based sensor, this study was not able to validate the RR measurement. For SpO_2_, however, this study is the first to report a moderate relationship between SpO_2_ from a wearable sensor and reference monitoring.

Although the biases for Radius PPG were within this threshold, the LoAs were wider than the predefined cutoff values in all vital signs. In contrast, Van der Stam et al. found a smaller mean difference and LoA for RR using Bland–Altman analysis of a PPG wristband. However, with an exclusion rate of 66% due to a low-quality index, the findings cannot be easily extrapolated [24]. Validity and reliability data of wearable sensors that measure SpO_2_ are scarce [25]. This study demonstrates mean differences of 0.4% (randomly selected pairs) and 0.6% (30-min pairs) for SpO_2_, which are acceptable. However, also for SpO_2_, LoA could not fit into the clinically acceptable value. Additionally, the median (IQR) results measured by Radius PPG were similar to the median (IQR) of the reference monitor, which results in a MAPE maximum of 1.6%. Contrary to these findings, our research group in a previous study found a MAPE of 5.9% for SpO_2_ compared with nurse measurements [22]. However, we reported a low availability of SpO_2_ in this previous study, which possibly influenced the results.

### 4.3. ECG-Based versus PPG-Based Wearable Sensors

Charlton et al. stated that algorithms based on ECG were typically more precise than those based on PPG [26]. In line with that, our findings showed that HR measured by the ECG-based sensor in the 30-min analysis were the only measurements considered reliable, e.g., mean difference and LoA were within the predefined clinically acceptable range. In contrast, the validity and reliability of the two wearable sensors were comparable in all other analyses. Besides measurement quality, other sensor characteristics must be taken into account before implementation in a surgical ward. Depending on the patient’s condition or pathology, a sensor that measures the appropriate parameters should be selected. Nowadays, nurses collect and interpret the parameters HR, RR, temperature, and SpO_2_. The ability to measure SpO_2_ with a wearable sensor can be clinically relevant. Especially in the case of high-risk postsurgical patients, SpO_2_, in combination with RR, serves as a crucial parameter for the detection of respiratory deterioration, which may occur as an adverse effect of anesthesia in the first 24 to 48 h after surgery. This parameter can only be measured non-invasively by PPG-based sensors. Other characteristics that should be taken into account are battery life, location on the body, technical feasibility, and patient satisfaction. This is an important addition to feature research since the implementation of wearable sensors only addresses the clinical challenges if patients and nurses are willing to adopt the technology.

### 4.4. Statistical Analysis

This study provides some analytic strategies for other researchers to assess vital sign data for validity and reliability. The use of these strategies was selected due to the absence of an established standard monitoring frequency for patients in surgical wards. Other studies use similar strategies. Van Rossum and colleagues used a minute-sampled vital sign recording and resampled it by averaging the signal values in multiple time windows [27]. Breteler and coworkers resampled the originally transmitted sensor data (once every 4 s) once every minute [21]. Contrary to our approach, they used one sample corresponding to the nearest time point of the reference monitor. The chosen analysis is important because it may influence the interpretation of the sensor’s performance, especially because cutoff values are based on expert opinion. For example, in the case of HR for the Masimo Radius PPG, there may be clinically relevant differences between the LoA, which range from −14.2 to 13.6 bpm (random four-point strategy) compared with −10.4 to 9.0 bpm (1-min median filter strategy). Whether it is clinically relevant must be determined by further research. Clark error grid analysis can be used to quantify the implications of the differences between the vital signs measured by the wearable sensor versus the reference monitor.

### 4.5. Implications

Although our study provides important information for this first step of implementation, our intended environment for using wearable sensors is not the PACU but the surgical ward. It is expected that patient movement influences sensor measurements to a larger extent in the ward compared to the PACU. The patients at the PACU included in this study were in bed with vital signs within normal ranges most of the time. At the surgical ward, increased patient movement and deviating vital signs induce higher variability in vital sign measurements, which may result in lower measurement validity and reliability of the sensors [11]. Even though measurement quality is one of the most important criteria for any patient monitoring technology, the choice for a wearable sensor should be a trade-off between measurement quality and a sensor’s usability for monitoring patients, enabling early mobilization at the ward for several days [9].

Furthermore, several challenges need to be addressed before incorporating wearable sensors into the work processes in the surgical ward. The focus should be shifted from spot measurements to trend monitoring because patients admitted to the surgical ward have an increased care burden, whereby early deterioration can easily be missed. To achieve early detection of deterioration or complications, automatic recognition of changes in vital signs is necessary. This will support healthcare professionals in clinical decision-making. For example, Van der Stam and colleagues developed a remote early warning score using vital parameters collected by a wearable sensor as a first step toward remote monitoring and data-driven decision support for patients undergoing major abdominal cancer surgery [28]. The diagnostic value of this method was comparable to the modified Early Warning Score. However, larger-scale follow-up is needed.

The increase in healthcare costs over the years is a common topic in worldwide discussions. However, knowledge of the clinical impact and cost-effectiveness of continuous vital signs monitoring is scarce. Vroman et al. assessed changes in clinical outcomes and in-hospital costs before and after the implementation of a wearable sensor in postsurgical patients. They found a negative impact on in-hospital costs, relating to a lower intensive care admission rate in the intervention group [29]. However, this study was designed as an observational pre-post analysis, whereas randomized controlled trials are needed. Overall, future research should focus on developing algorithms based on continuous measurements for supporting healthcare professionals and conducting evidence of the effects of continuous vital signs monitoring on clinical and financial outcomes.

### 4.6. Strengths and Limitations

The strengths of the present study include its design evaluating two types of sensors in a clinically relevant practice. The implementation of wearable sensors is proposed as a potential solution to limited health staff challenges and the increasing care burden. However, for this to be effective, robust comparisons with a reference monitor must first be performed. This study provides such comparisons.

The PACU is the most suitable setting for such a study because patients are connected to the bedside monitor while awake. A disadvantage is that reference monitoring at the PACU cannot be considered the gold standard for RR and SpO_2_, which are manual counting or thoracic impedance and an arterial catheter, respectively. A limitation of this study is that unknown measurement errors of the reference device were not taken into account in interpreting the results as Breteler et al. did by using mixed-effect models [23]. We also used relatively strict acceptable limits for mean difference and LoA compared to previously reported data by Leenen et al., being 10 ± 10 bpm for HR, 3 ± 3 brpm for RR, and 3 ± 5% [30]. Both may have led to lower reliability than the actual reliability of the sensors.

The study protocol did not include the addition of a urinary catheter to the standard of care, which is a limitation of this study. In patients without a urinary catheter, the standard temperature was measured by nurses using a temporal scanner (Exergen Corporation, Watertown, MA, USA). However, the temporal scanner is not recommended for use as a reference [31,32]. Owing to the low number of patients in whom a temperature assessment was possible (VitalPatch, n = 5; Radius PPG, n = 12), no statistical analyses were performed.

Another limitation of this study is inadvertent data loss. Data loss in VitalPatch was mainly caused by the action required for the calibration of temperature 30 min after the sensor was applied. Consequently, no temperature readings were available before calibration, which is certainly not desirable, particularly in the initial postoperative phase. Another reason for data loss in VitalPatch was connectivity problems. Difficulties were experienced several times in connecting VitalPatch using Bluetooth. For Radius PPG and Radius T, the main reason for data losses was problems with exporting the data. Data were stored locally at the bedside monitor with the limited data storage capability of 96 h, causing the first data to be overwritten after this period. Avoiding the need for calibration and transferring data in real time using WiFi may reduce these types of data loss.

## 5. Conclusions

In conclusion, while HR, RR, and SpO_2_ have mean differences within acceptable cutoff values for both wearable sensors, only ECG-based HR measurements reached the LoA thresholds and were considered clinically reliable. This study demonstrated the validity for HR and SpO_2_ measurements in both sensors but not for RR in either sensor. The validity and reliability of vital sign measurements by wearable sensors influence the interpretation of data and, consequently, clinical decision-making. Providing information on the performance of various wearable sensors compared to a standard clinical reference monitor in the target population is, therefore, important and will support the implementation of wearable sensors into the work processes on the surgical ward.

## Figures and Tables

**Table 1:** Patient characteristics.

Patient Characteristics	Data Value
	(N = 58)
Age, median (IQR), years	55.5 (41.5–66.8)
Sex, n (%)	
Male	33 (57)
Female	25 (43)
American Society of Anesthesiologists Physical Status, n (%)	
I	14 (24)
II	31 (54)
III	13 (22)
IV	0 (0)
Emergency admission, n (%)	
Elective	31 (54)
Nonelective	27 (46)
Type of surgery	
Upper extremities	6 (10)
Lower extremities	21 (36)
Acetabulum	7 (12)
Spine	13 (23)
Infection	7 (12)
Other	4 (7)
Length of stay	
Hospital, median (IQR), days	12 (6–18)
Postoperative, median (IQR), days	7.5 (4–14)
PACU, median (IQR), hours	2.2 (1.5–3.1)

**Table 2:** Number of available data pairs, median values, interquartile ranges, and median absolute percentage error for VitalPatch compared to reference measurements per vital sign.

Vital Sign			Reference Monitor	VitalPatch	
	No.	Pairs	Median (IQR)	Median (IQR)	MAPE (%)
Heart rate (bpm)					
4 points	49	196	82.5 (70.5–92.0)	82.5 (70.0–92.5)	3.8
30 min	49	1356	83.0 (68.5–92.0)	83.0 (69.0–92.0)	1.7
Respiration rate (brpm)					
4 points	43	172	15.0 (12.0–18.0)	15.0 (13.0–19.0)	22.8
30 min	43	1194	15.0 (12.5–17.0)	15.0 (12.0–18.0)	20.8
Temperature (°C)					
4 points	5	20	37.3 (37.1–37.5)	37.3 (36.8–37.5)	0.8
30 min	5	136	37.3 (37.0–37.4)	37.2 (36.7–37.3)	0.8

No.: number of patients; IQR: interquartile range; MAPE: median absolute percentage error; bpm: beats per minute; brpm: breaths per minute; °C: degrees Celsius.

**Table 3:** Number of available data pairs, median values, interquartile ranges, and median absolute percentage error for Radius PPG and T wearable sensors compared to reference measurements per vital sign.

Vital Sign			Reference Monitor	Radius PPG and T	
	No.	Pairs	Median (IQR)	Median (IQR)	MAPE (%)
Heart rate (bpm)					
4 points	51	204	83.0 (70.0–91.0)	82.0 (70.0–91.0)	5.2
30 min	51	1453	83.0 (70.0–92.0)	81.0 (69.0–92.0)	2.5
Respiration rate (brpm)					
4 points	51	204	16.0 (12.0–19.0)	14.0 (12.0–17.0)	20.4
30 min	51	1400	15.0 (12.5–17.5)	14.0 (11.0–17.0)	19.0
SpO_2_ (%)					
4 points	51	204	96.9 (95.3–98.0)	97.0 (96.0–99.0)	1.3
30 min	51	1454	97.2 (95.3–98.0)	97.0 (96.0–99.0)	1.1
Temperature (°C)					
4 points	12	48	37.1 (36.7–37.4)	36.5 (36.3–36.6)	1.5
30 min	12	349	36.9 (36.7–37.3)	36.6 (36.4–36.6)	1.5

No.: number of patients; IQR: interquartile range; MAPE: median absolute percentage error; bpm: beats per minute; brpm: breaths per minute; SpO_2_: blood oxygen saturation; °C: degrees Celsius.

**Table 4:** Mean difference and 95% limits of agreement for VitalPatch and Radius PPG wearable sensors compared to reference measurements per vital sign.

Vital Sign	VitalPatch			Radius PPG		
	No.	Pairs	Mean Difference (95% LoA)	No.	Pairs	Mean Difference (95% LoA)
Heart rate (bpm)						
4 points	49	196	0.0 (−9.0–9.1)	51	204	−0.3 (−14.2–13.6)
30 min	49	1356	0.2 (−4.6–5.0)	51	1453	−0.7 (−10.4–9.0)
Respiration rate (brpm)						
4 points	43	172	0.6 (−8.2–9.3)	51	204	−1.3 (−9.6–7.1)
30 min	43	1194	0.4 (−7.2–8.0)	51	1400	−0.6 (−7.9–6.7)
SpO_2_ (%)						
4 points	-	-	-	51	204	0.6 (−2.4–3.6)
30 min	-	-	-	51	1454	0.4 (−2.3–3.1)

No.: number of patients; LoA: limits of agreement; bpm: beats per minute; brpm: breaths per minute; SpO_2_: blood oxygen saturation.

## Data Availability

The datasets presented in this article are not readily available because the data are part of an ongoing study.
